# An Autobiographical Case Report on Papillary Thyroid Carcinoma with Positive Antithyroid Antibodies: Coincidence or Correlated?

**DOI:** 10.7759/cureus.44651

**Published:** 2023-09-04

**Authors:** Arkaja Singh, Sameer Rao, Dev Yash Rana, Mrinal Choudhary, Romil Singh

**Affiliations:** 1 Medicine, Mahatma Gandhi Medical College and Hospital, Jaipur, IND; 2 Gastroenterology and Hepatology, Mayo Clinic, Rochester, USA; 3 Medicine, Sawai Man Singh (SMS) Medical College, Jaipur, IND; 4 Internal Medicine, JSS Medical College, Mysuru, IND; 5 Medicine, JSS Medical College, Mysuru, IND; 6 Critical Care, Allegheny Health Network, Pittsburgh, USA

**Keywords:** hashimoto's thyroiditis, autoimmunity, antithyroid antibodies, tsh, thyroid cancer, papillary cancer of thyroid

## Abstract

The relationship between autoimmunity and cancer has been a gray area, with many theories but no solid proof so far. Hashimoto's thyroiditis is an autoimmune disorder and a major cause of hypothyroidism, while papillary thyroid carcinoma is the most common thyroid malignancy generally found in patients younger than 45 years of age. The literature on the correlation between these two disorders is somewhat based on potentially biased histopathological examination from pre-operative fine needle aspiration and post-thyroidectomy samples. Although recent studies are evaluating a possible holistic molecular, hormonal, and histopathological foundation for this correlation, a clear causal relationship has not been established yet. This report illustrates the author's case presentation, treatment, and eventual outcome of the disease when she was diagnosed with papillary thyroid cancer at the age of 25 years, with positive antithyroid peroxidase and antithyroglobulin antibodies.

## Introduction

The disease forms of thyroid carcinomas vary from commonly found slow-growing circumscribed papillary thyroid cancer (PTC) to rare fulminant and fatal anaplastic thyroid cancer. The spectrum of thyroid carcinomas includes papillary, follicular, medullary, and anaplastic in order of descending prevalence. The predominant cell type in the thyroid gland is follicular cells, which originate from the endoderm and form thyroid follicles, which are the structural and functional elements of the thyroid gland. In between these follicles are parafollicular cells (C cells), which are derived from the neural crest and secrete a hormone known as calcitonin. Different forms of cancer result from the transformation of these parafollicular cells. Papillary and follicular thyroid carcinoma are the two primary types of differentiated thyroid cancer derived from follicular cells. Anaplastic and poorly differentiated thyroid carcinomas are uncommon tumors that develop from follicular cells and have a poor prognosis. The classic C-cell tumor is medullary thyroid carcinoma, with unique biologic characteristics such as a weakly defined, infiltrative tumor with discohesive cell nests embedded in a fibrous stroma that may contain amyloid [1,2].

Approximately 85% of thyroid malignancies are PTC. Between 1975 and 2009, the incidence of papillary thyroid cancer in the United States quadrupled, owing primarily to the accidental discovery of small-volume papillary carcinomas during imaging investigations. The absolute increase in thyroid cancer incidence in women was almost four times greater than that of men. The mortality rate attributable to thyroid cancer is low and has shown stability from 1975 to 2009, with approximately 0.5 deaths per 100,000 patients [3].

Thyroid cancer is often diagnosed incidentally as it commonly manifests as an asymptomatic nodule, making it difficult to diagnose without it invading or spreading to other body areas causing symptoms [4]. Hashimoto's thyroiditis (HT) is a prevalent contributor to hypothyroidism, notably in regions with adequate iodine levels. This condition involves T-lymphocytes' infiltration of the thyroid gland, resulting in the destruction of thyroid follicles and substituting these tissues with fibrous tissue. Diagnostic markers for HT comprise the existence of antithyroid autoantibodies (directed against thyroid peroxidase (anti-TPO) or antithyroglobulin (anti-Tg)) and an appearance of thyroid ultrasound as hypoechoic and heterogeneous, often with pseudonodular hypoechoic infiltration [5,6]. HT may result in persistent inflammation, which could contribute to developing thyroid cancer. However, the precise connection remains unclear, underscoring the importance of further extensive research to either confirm or challenge this association.

The management of PTC is primarily surgical. For individuals diagnosed with differentiated thyroid cancer (DTC) at stage T1b-T2, with tumors sized 1-4 cm and no signs of extrathyroidal extension (ETE) or lymph node metastases based on clinical or radiographic evaluation, the advisable primary surgical approach is either a thyroid lobectomy (TL) or a total thyroidectomy (TT). The available data suggest that prophylactic central neck dissection (pCND) should be contemplated for patients with advanced primary thyroid tumors (T3 or T4) because they carry a notable risk of locoregional recurrence, which can be reduced through pCND [7].

This report details the author's experience receiving a PTC diagnosis at the age of 25, including the commencement of the symptoms, the accompanying workup, and the subsequent course of therapy.

'I' will refer to myself, Arkaja Singh. Through this case report, I convey my journey as a physician who was left flabbergasted by an unexpected cancer diagnosis and transitioned from being a doctor to a patient in the blink of an eye. I hope this case report will help people diagnosed with cancer understand the disease and what they can expect in the course of the disease. I will also briefly discuss the relationship between autoimmunity and cancer and how it could be a potential research area for better understanding the pathophysiology of thyroid cancer.

## Case presentation

**Figure 1:**
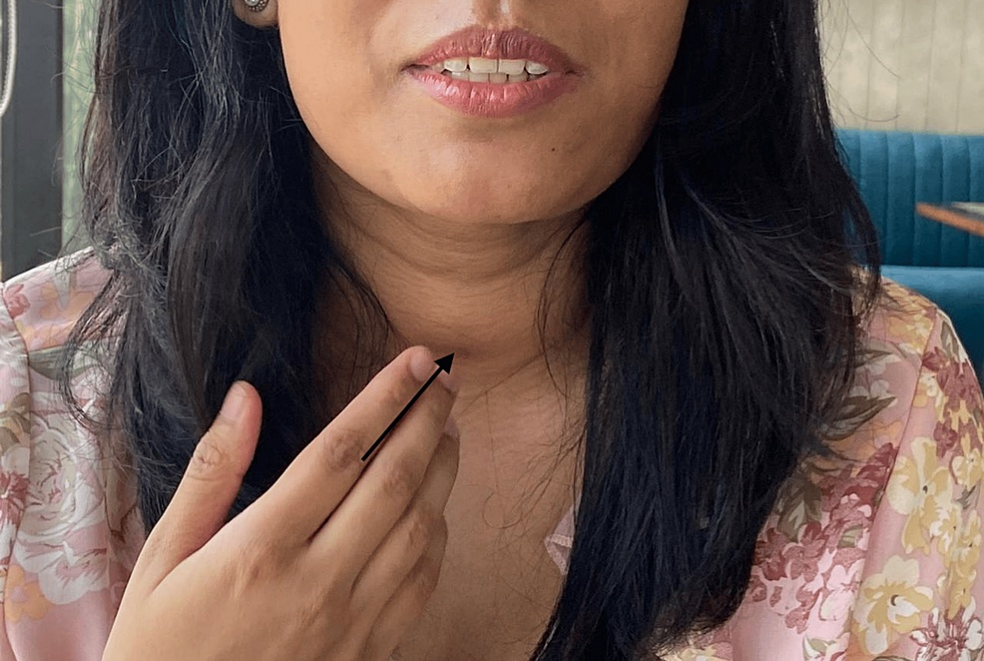
Initial presentation of small midline neck mass which was more visible on swallowing.

It was the month of May 2023; I was fresh out of medical school, a graduate, and a doctor. I was a month away from taking my United States Medical Licensing Examination step 2. I was eating healthy and exercising right and had self-managed my polycystic ovarian disease with lifestyle changes for the past four years. One day I went out with a friend to take a break from the usual schedule when he suddenly pointed out a minute swelling on my neck. It felt like a hard lump; nothing particularly important occurred to me as I tried to come up with as many differentials as possible. I was standing in front of the mirror trying to see what this swelling was and noticed it moved on deglutition (Figure 1). Like every busy student, I ignored the swelling for a week and took things lightly as I have no family history of thyroid pathologies. As I came out of my denial period, this was when I thought maybe this was not that simple. Little did I know that there was something else planned for me. I got my thyroid profile (TSH, T3, and T4) done. The results were within the normal range, which was very much expected, given my negative family history. I consulted my primary care physician, who attributed the swelling to a goiter caused by iodine deficiency. This explanation struck me as odd, given my sufficient iodine intake and the sudden onset of the swelling. Trusting my instincts, I sought a second opinion. The recommendation was to undergo a neck ultrasound and a thyroid antibody profile to ensure I covered all bases.

Findings

Thyroid function testing has limited utility in diagnosing PTC because most patients have normal thyroid function, which was the case in my situation as well. TSH and free T4 levels within the normal range are typical in thyroid cancer patients [8]. Thyroid antibody testing was positive for antithyroid peroxidase (263.66 IU/ml) and antithyroglobulin antibodies (148.91 IU/ml).

**Figure 2:**
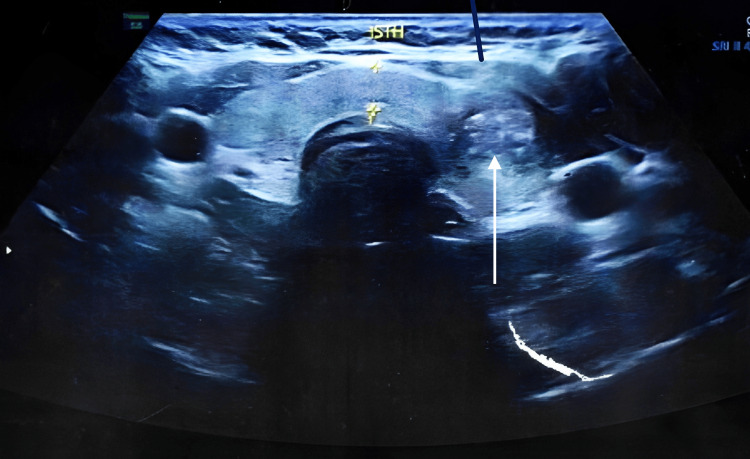
USG of the neck showing thyroid nodule in the left lobe near the isthmus, and calcifications all around the nodule.

**Figure 3:**
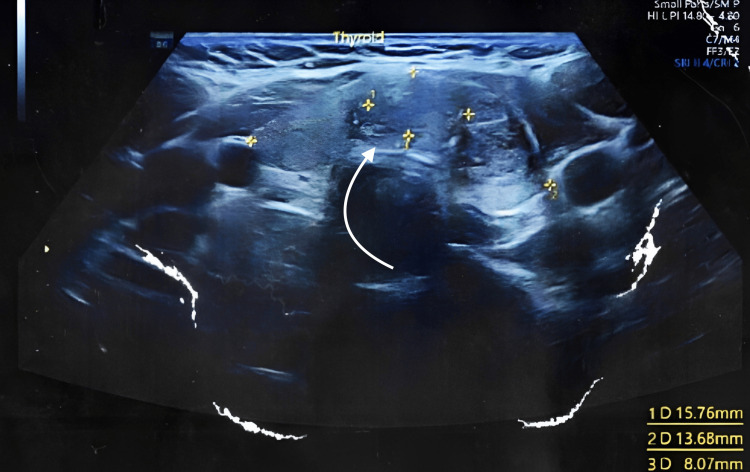
USG of the neck showing thyroid nodule of dimensions of around 15.76x13.68x8.07mm.

Ultrasound of the neck found a multifocal echogenic foci lesion with poor vascularity (Figures 2-3). The lesion size was 15.76x13.68x8.07mm in the left lobe near the isthmus. It was classified as Thyroid Imaging Reporting and Data Systems (TIRADS) V, and I was advised to get a fine needle aspiration cytology (FNAC) of the thyroid nodule.

FNAC findings included scattered papillary sheets and clusters of mild pleomorphic follicular cells, scattered foamy macrophages, a few multinucleated giant cells, occasional epithelioid cell-like cells, and scanty colloid material. Intranuclear inclusions·and nuclear grooving were also seen. These findings were consistent with papillary carcinoma (Bethesda class V). 

A contrast-enhanced MRI was done before the surgery, which showed no tracheal involvement and no obvious lesion on the tongue, oropharynx, vallecula, pyriform sinuses, and glottic and subglottic larynx. It did show slight bilateral enlargement of level IA, IB, AND II cervical nodes of unknown significance. 

Within two weeks of everything, I was standing with all the reports in my hand and shaken to the core, but I had to keep a smile on as my family was more petrified than I was. My mind was baffled about how this could have happened to me, but I was fortunate to have supportive family and friends. During that time, I truly realized how vital social support and reassurance are while dealing with a condition like cancer. I was asked to schedule my surgery as soon as possible. 

Surgery

TT with central compartment clearance and bilateral neck dissection was done, keeping the common spread to lymph nodes in mind. Though the ideal management for the stage of cancer I had should have been either a TL or a TT, the surgeons were concerned about the MRI finding of slight bilateral enlargement of level IA, IB, and II cervical nodes of unknown significance, which led them to take this radical approach. 

**Figure 4:**
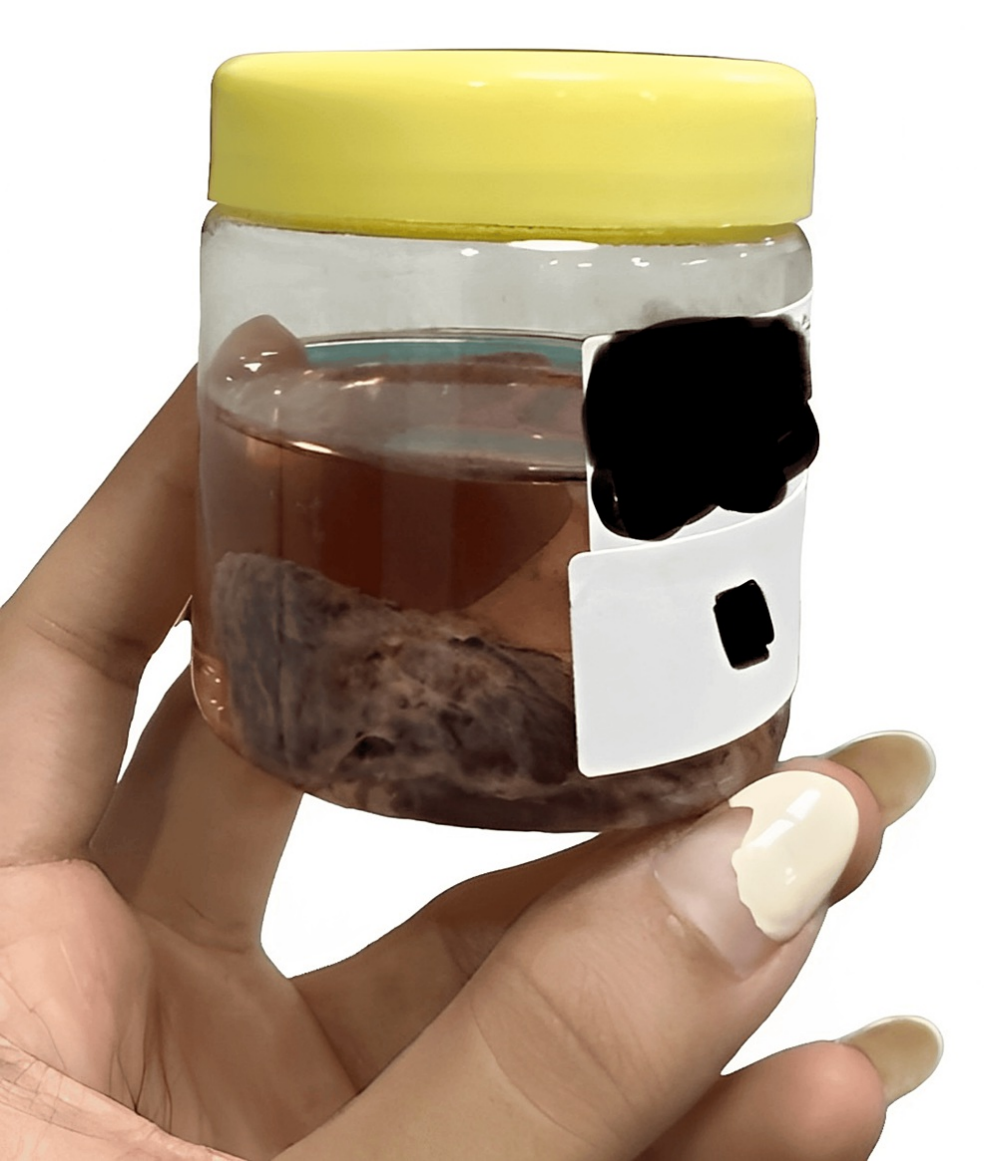
Gross specimen of the left thyroid lobe.

**Figure 5:**
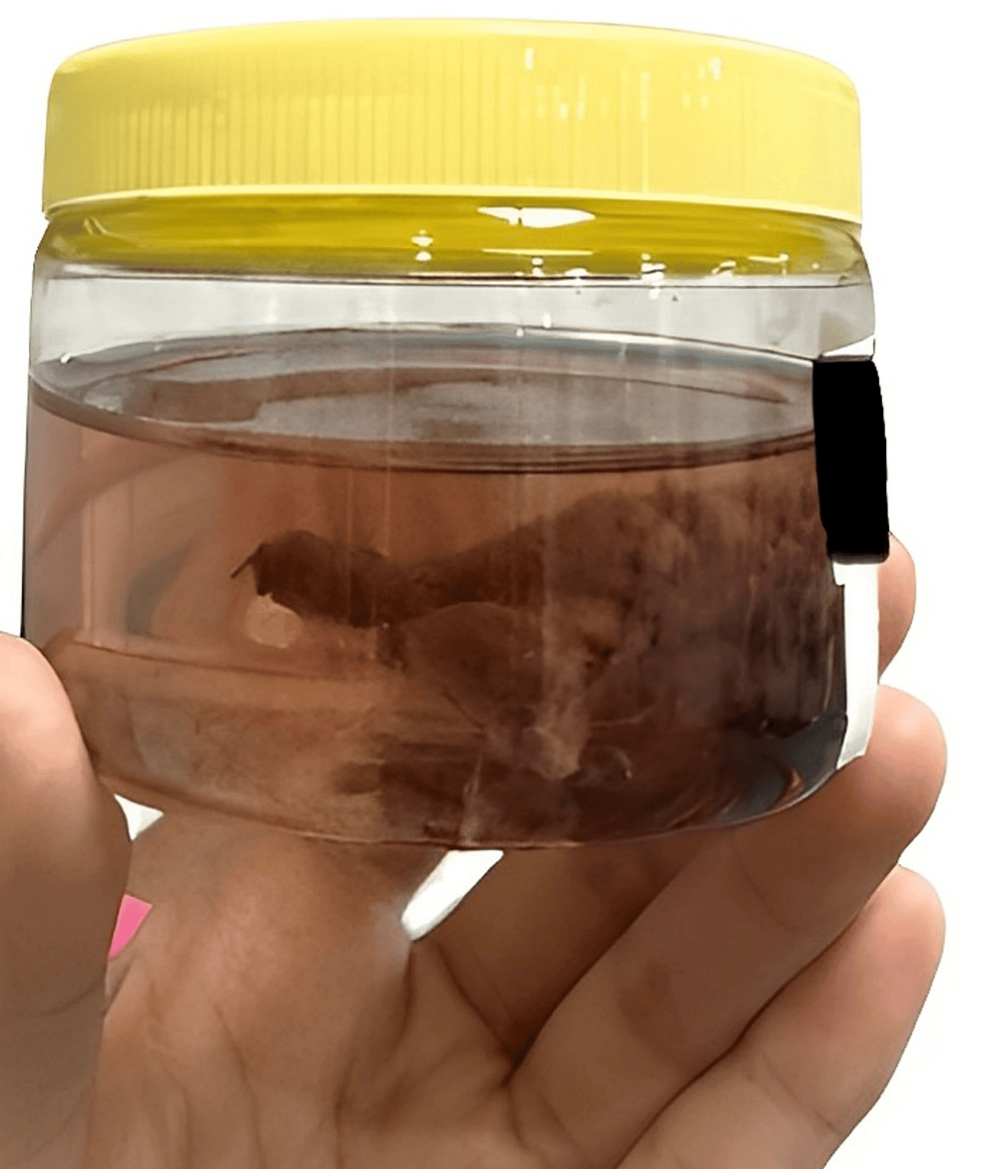
Gross specimen of the right thyroid lobe.

**Figure 6:**
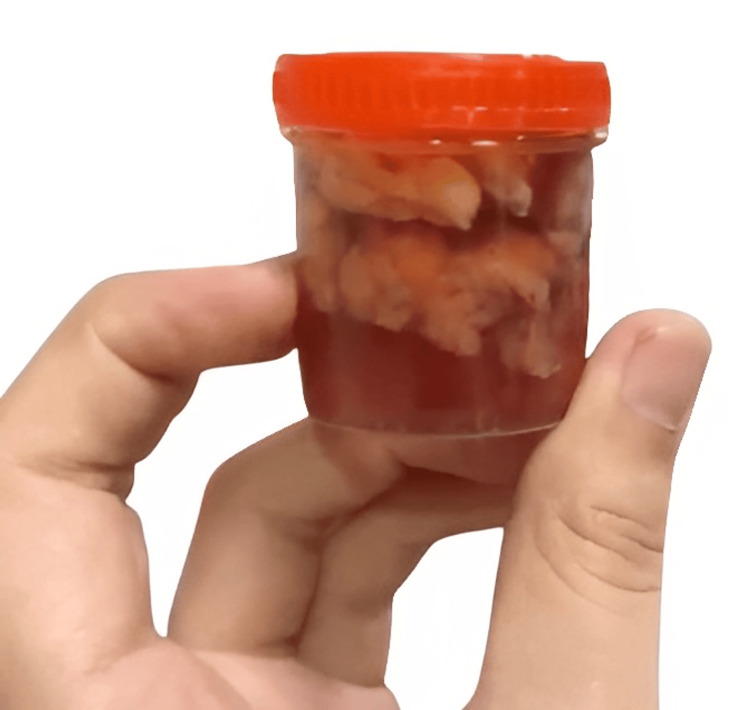
Gross specimen of the lymph nodes removed from levels II, III, IV.

Intraoperative findings included an enlarged left lobe of the thyroid measuring 5 x 4 cm, characterized as hard and nodular (Figure 4). The right thyroid lobe was 2 x 2 cm in dimensions (Figure 5). Additionally, a prominent pyramidal lobe was observed. A small, round, discrete lymph node was noted in the left paratracheal region. Multiple small, round, discrete nodes measuring 2 x 1 cm were observed along levels III and IV on the left side. Similarly, nodes of size 2 x 1 cm were noted along levels III and IV on the right side. The removed thyroid and lymph nodes were sent for histology. The final staging was determined to be pT2N0, indicating that the tumor was in stage 2 and no regional lymph node metastasis was observed. The margins were clean, and the lymph nodes showed no signs of malignancy (Figure 6).

Postoperative treatment, radiation, and long-term follow-up

Initially, after the surgery, I developed transient hypocalcemia which was corrected in a few days with calcium supplements. I did not develop any hoarseness, indicating no injury to recurrent laryngeal nerves. 

I found an area over my right shoulder to be numb with no sensations and weak overhead abduction of my right shoulder. I went to an orthopedician who suggested physiotherapy and pregabalin for my neuropraxia. Although my symptoms improved, they did not completely resolve. Neuropraxia is a mild form of nerve injury that usually occurs due to trauma. It is characterized by transient injury to the myelin sheath without disruption of axon continuity. It causes temporary paresthesia or weakness as a result of blocked nerve transmission. Electromyography shows fibrillations and reduced motor unit action potentials [9]. The use of electrocautery in surgery to reduce the bleeding risk can lead to damage to peripheral nerves, which could be the potential cause of neuropraxia in my case [10]. 

**Figure 7:**
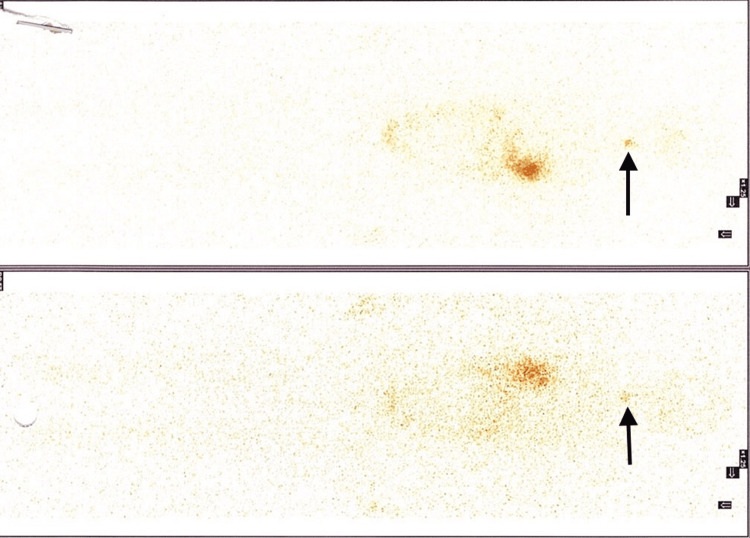
Whole-body radioactive iodine uptake assay showing remnant thyroid tissue at the base of the thyroid gland.

After 15 days, a whole-body radioactive iodine assay was done to see if any remnant tissue or extrathyroidal tissue was present (Figure 7). The results showed that a small amount of thyroid tissue was left at the base of the thyroid gland. Radioactive Iodine ablation (30 mCi) was given, and I was told to isolate for one week with low iodine intake for better treatment outcomes. I was started with 125 mcg of thyroxine to suppress thyroid stimulating hormone (TSH) and advised to get a thyroid profile done every four weeks for at least six months. Given the high recurrence of this cancer, my doctor suggested that I get regular checkups and scans every six months for the first two years and then yearly for the next five years.

## Discussion

While recuperating from my surgery, I reviewed my investigation reports and contemplated the elevated levels of my antithyroglobulin antibodies (TgAb) and anti-thyroid peroxidase (TPO) antibodies (TPOAb). As a curious clinician, I felt compelled to delve into the underlying reasons. My curiosity led me to explore numerous research articles, revealing a complex and little-understood correlation between autoimmunity and cancer development. Despite being considered and studied as separate pathologies for decades, these two conditions might not be as different as once thought. Immune tolerance is a process by which the immune system does not respond to self-tissues while still being able to recognize and react to harmful antigens. Central tolerance is the mechanism through which the body controls this process and eliminates the rule breakers using the Treg, Breg, and M2 macrophages. However, these cells are reduced in some people, and they escape the central tolerance causing autoimmunity. On the other hand, cancer cells can escape the immune system by mechanisms such as limiting the detection of antigens, suppressing the immune system, and triggering T-cell exhaustion. To counter this, researchers have developed anti-cancer drugs that recognize the immune checkpoints and target the PD-1 (programmed cell death receptor 1) and CTLA-4 (cytotoxic T lymphocyte antigen 4) pathways demonstrating the value of researching autoimmune diseases in the creation of new anti-cancer medications and shed some light on the hypothesis immune tolerators like PD-1, IL-10, and adenosine are increased. However, with autoimmune diseases, these immune tolerators and regulatory cells decrease [11].

Thyroid cancer can potentially be regarded as an outcome of HT, owing to the role of chronic inflammation in cancer development. The underlying pathology behind this hypothesis is relatively straightforward: chronic inflammation induces cell injury, leading to the accumulation of reactive oxygen species (ROS) and attracting inflammatory cells, chemokines, cytokines, and growth factors for cell repair and proliferation. The ROS generated during inflammation also causes DNA damage, a significant driver of cancer development. Moreover, the chemokines, cytokines, and growth factors known for promoting cellular transformation and tumor progression contribute to the process as well [12]. HT occasionally displays nuclear changes, including RET/PTC rearrangements and BRAF mutations, which bear similarities to those observed in PTC and imply that the neoplastic and autoimmune diseases share the same molecular pathophysiology [13]. In cases of HT coexisting with PTC, RET/PTC rearrangement, producing a persistently activated receptor with tyrosine kinase activity, is more commonly identified. Interestingly, benign tumors, particularly HT, have also shown RET/PTC rearrangements. One theory posits that the inflammatory environment of HT could heighten the probability of RET/PTC rearrangement, subsequently increasing the risk of additional DNA abnormalities that ultimately culminate in thyroid cancer [14]. Daniel Lubin et al. conducted a research study to better understand molecular changes associated with this correlation. They found statistically significant results with PD-L1 expression in HT and the development of PTC in the background [15]. 

Numerous research studies have found an association between HT and PTC. One study showed a greater incidence and risk of PTC in HT patients, especially males. Along with that, it was seen that patients with coexisting HT and PTC had less invasive disease. A similar trend emerged in another study, where they drew a resemblance of the role of HT in PTC to a 'double-edged sword'. Their meta-analysis revealed that while HT increases the risk of PTC, the extent of spread beyond the thyroid, as well as metastasis and recurrence, was notably reduced in patients with coexisting PTC and HT [16]. Another piece of research supported this notion of HT acting as a protective factor in PTC cases. They observed that patients with coexisting PTC and HT exhibited better 10-year disease-specific survival rates and lower mortality related to PTC [17]. 

Although I was never formally diagnosed with HT, the presence of antithyroid antibodies such as antithyroglobulin and TPOAb, and my histopathology report having signs indicative of thyroiditis such as the presence of giant cells with limited colloid, it is clear that I was a patient with asymptomatic HT. Several studies have noted a connection between antithyroid antibodies and PTC. A retrospective cross-sectional study found that positive TPOAb could be an independent predictor of thyroid malignancy, increasing the risk to 2.28 times compared to seronegative patients [13]. Another study evaluated the role of these antibodies with the progression of PTC, revealing that elevated levels of pre-operative antithyroglobulin antibody (TgAb) could be an independent predictor of lymph node and extranodal extension. Additionally, levels of TPOAb were observed to be inversely proportional with pathologic T- and N- stages [18]. All these studies point toward some correlation between HT and PTC. At this juncture, comprehending how long I have had asymtomatic HT, which may have been a leading factor in cancer development as I did not have any other risk factors, remains challenging. Although this correlation between HT and PTC has been discussed in multiple research articles, a precise underlying mechanism still has not been understood. I am hopeful that my case will help in illuminating this enigmatic territory.

## Conclusions

While a significant number of PTC patients are diagnosed after it has spread to regional lymph nodes, I consider myself fortunate to have detected papillary thyroid carcinoma (PTC) at an early stage. The prognosis of coexisting HT and PTC appears more favorable than age- and stage-matched PTC cases alone. Despite this, the current therapy for PTC with or without coexisting HT remains identical. PTC patients with autoantibodies necessitate a more intensive diagnostic and follow-up approach, underscoring the importance of expanding our knowledge and literature on the intricate relationship between autoimmunity and thyroid cancers. Through this case report, we bring a unique perspective of a young physician dealing with a sudden diagnosis of thyroid cancer, which led her to explore the literature to find any potential explanation for how this could have happened to her. The interplay between autoimmunity and cancer, i.e., HT and PTC appeared to her as one potential suspect. This is a very commonly seen correlation that beckons further investigation to comprehend the exact pathology lying within the convergence of these two facets of the same coin.
